# Biocosmetics Made with *Saccharina latissima* Fractions from Sustainable Treatment: Physicochemical and Thermorheological Features

**DOI:** 10.3390/md21120618

**Published:** 2023-11-29

**Authors:** Noelia Flórez-Fernández, Tania Ferreira-Anta, Julie Queffelec, Isa B. Ingrez, Manuela Buján, Antonio Muiños, Herminia Domínguez, María Dolores Torres

**Affiliations:** 1 CINBIO, Chemical Engineering Department, Faculty of Science, Campus Ourense, Universidade de Vigo, As Lagoas S/N, 32004 Ourense, Spain; noelia.florez@uvigo.gal (N.F.-F.); tania.ferreira@uvigo.gal (T.F.-A.); julie.queffelec@uvigo.gal (J.Q.); matorres@uvigo.gal (M.D.T.); 2 Portomuíños, Polígono Industrial, Rúa Acebedo, Parcela 14, Cerceda, 15185 A Coruña, Spain; imasd1@portomuinos.com (I.B.I.); matorres@vigo.gal (M.B.); antonio@portomuinos.com (A.M.)

**Keywords:** antioxidants, brown seaweed, cold cream, microparticles, ultrasound-assisted extraction, viscosity

## Abstract

This work deals with the formulation of natural cosmetics enriched with antioxidant fractions from the ultrasound treatment (US) of the brown seaweed *Saccharina latissima*. The challenge was the development of a cosmetic matrix without jeopardizing the thermorheological features of the creams, adding microparticles containing the antioxidant fractions using two different carriers, mannitol and alginate. The fundamental chemical characteristics of seaweed and the extracts obtained *via* sonication, as well as the antioxidant properties of the latter, were analyzed. The highest TEAC (Trolox equivalent antioxidant capacity) value was identified for the extracts subjected to the longest processing time using ultrasound-assisted extraction (240 min). A similar yield of microparticle formulation (around 60%) and load capacity (about 85%) were identified with mannitol and alginate as carriers. Color testing of the creams exhibited small total color differences. The rheological results indicated that the testing temperature, from 5 to 45 °C, notably influenced the apparent viscosity of the matrices. All creams were adequately fitted with the two parameters of the Ostwald–de Waele model, with the flow consistency index following an Arrhenius dependency with the testing temperature. Neither hysteresis nor water syneresis was observed in the proposed cosmetics during 6 months of cold storage at 4–6 °C.

## 1. Introduction

Brown seaweeds, such *Saccharina latissima*, have biomedical and cosmetic potential due to their biological properties, which include antitumoral, antidiabetic, anti-inflammatory, antioxidant, antibacterial and prebiotic characteristics, among others [1,2,3]. Additionally, properties associated with the field of cosmetics have been also found; photoprotection, anti-aging, anti-melanogenic and antimicrobial properties are some of them [4]. These characteristics have been associated with fucoidan, a polysaccharide comprising mainly fucose and sulphate groups but with other saccharides also present in its structure [4,5,6,7]. Brown seaweeds have other molecules with biological potential, such as phlorotannins, pigments, alginate and laminarin, among others [8,9,10,11,12].

In order to obtain these bioactive compounds from algae, extraction techniques are the way to recover them [13]. The selection of an adequate extraction technology is important to obtain a crude extract, a subject of interest to several industries. Currently, in line with the concept of sustainability, the use of ecofriendly technologies, such as ultrasound-assisted extraction, hot pressurized water extraction and microwave-assisted extraction, have been proposed in several research works, using water as a solvent [14,15,16,17,18]. Ultrasound-assisted extraction is a technology that is widely used to obtain bioactive compounds from seaweeds [19]. Based on the principles of this technology, several parameters, such as solid liquid ratio, temperature, frequency and power, must be defined in an experimental work to achieve the desired product [20]. Lourenço-Lopes and coauthors (2023), focusing on the extraction of fucoxanthin, the main pigment in brown algae, explored ultrasound-assisted extraction and microwave-assisted extraction to obtain this valuable fraction from *Undaria pinnatifida* [21]. Based on the biorefinery concept, several fractions with potential research interests can be obtained from brown seaweeds [22].

According to the literature, the crude extracts obtained from seaweeds, and also the purified fractions, exhibit a variety of the aforementioned biological properties, making them suitable for applications in several fields, such as the food, pharmaceutical or cosmetics industry. Currently, industries are moving to formulate greener products, and seaweed is one of the available sources in nature with potential active ingredients [23]. Additionally, the use of natural sources allows for the replacement of synthetic compounds, achieving sustainable formulations [24,25]. In the human body, the skin is the largest organ and has important functions; it can also be considered as a drug delivery route, as delivery is non-invasive and allows for the direct application of the active compound. At present, this organ is subject to the effects of the environment, since it is exposed to air pollution and/or UV radiation, which generate free radical reactions that cause oxidation, associated with wrinkling, dehydration, elastosis, photo-ageing and pigmentation, among others.

Regarding application, crude extracts from brown seaweeds can be used to formulate polymeric microparticles to use as drug delivery systems [26]. Moreover, the selection of a suitable biopolymer (e.g., alginate, mannitol or carrageenan) allows for the production of microparticles with tailored characteristics and performances [27]. Examples in the cosmetic field have been found; Lebeer et al. (2018) produced a skincare product to modulate and reduce the symptoms of acne, using alginate as the carrier [28]. Costa and coauthors (2023) formulated biogenic silica microparticles with a non-cytotoxic effect on skin keratinocytes and anti-ageing and regenerative properties [29]. Schiavon et al. (2019) produced cosmetics with microparticles and added natural extracts, providing a photoprotective capacity and antioxidant and anti-ageing features [30].

Their appropriate features permit the use of these particulate systems, when incorporated into cosmetic creams, for skin drug delivery application. The assessment of the thermorheological features of the corresponding cosmetics-forming matrices is essential, not only to develop final products with adequate and suitable characteristics, but also during processing, manufacturing and storage [31]. The apparent viscosity of the prepared matrices is a relevant parameter for the selection of the correct processing stage and to improve the quality and shelf life of the end product [32,33]. Monitoring apparent viscosity at different temperatures and shear rates can give us information not only on the stability of cosmetics during storage (at low frequencies) but also on their ability to spread on the skin at high shear rates. One main target is to achieve functional matrices with tailored rheological characteristics suitable for cosmetic applications made in a sustainable way.

*Saccharina latissima* is an edible seaweed with potential research interest, due to its biological properties, associated mainly with the polymer known as fucoidan, and also to the phlorotannin content [34]. Therefore, polymeric microparticles could be formulated to protect and maintain the properties of these bioactive compounds. Two carriers, namely alginate, a polymer extracted from brown seaweeds, and mannitol, a polymer used in the pharmaceutical field as an excipient, were assessed to study the morphological characteristics and the rheological properties of the final product formulated with polymeric microparticles.

The main aim of this work was the development of cosmetic creams containing fucoidan, extracted from *Saccharina latissima* using ultrasound. The fucoidan fraction selected was incorporated into the cosmetics as polymeric microparticles, formulated with two carriers, mannitol and alginate, separately. The monitoring of the thermorheological behavior of the prepared natural creams was studied.

## 2. Results and Discussion

### 2.1. Raw Material

*Saccharina latissima* was the raw material used in the current work. The proximal composition is summarized in Table 1. The highest fraction was the carbohydrate content, around 52%, making it the most abundant glucose. Formic acid accounted for 1%; these results could be associated with the products of degradation, due to the severity of the hydrolysis process used to analyze the raw material [35].

Other studies also analyzed the composition of *S. latissima* harvested in Atlantic Canada [34]. These authors found similar values for carbohydrates (48%) and a higher quantity of protein (9%); this difference could be due to the location of the cultivation of the raw material. On the other hand, other authors studied the ash content of material cultivated between June and September. They found that the range of values was from 15% to 20%; these values are consistent with the current work [36]. Regarding the carbohydrate content, Nguyen and colleagues (2020) found that the glucose content was higher than the other saccharides present in *S. latissima* [37]. Additionally, Bogolitsyn and coauthors studied the seasonal variations in several brown seaweeds in the Artic, in two locations. In both locations, they found that the content of glucose was the maximum for *S. latissima* [36]. The analysis of the mineral fraction showed that the highest quantity was found for potassium; the same results were found in another work, where the same seaweed, but from another location, was analyzed [34].

### 2.2. Extraction Technology: Ultrasound-Assisted Extraction

**Figure 1:**
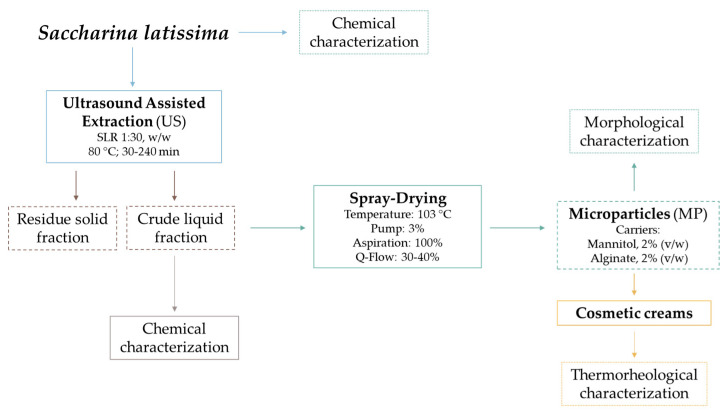
Flow diagram of the extraction process and the formulation of the microparticles to produce the final product, the cosmetic cream.

In the current work, the extraction technology used to obtain bioactive fractions was ultrasound-assisted extraction; Figure 1 shows the flow diagram of the extraction process carried out and the operating conditions, using *S. latissima* as the raw material, resulting in the formulation of cosmetics with polymeric particulate systems as microparticles.

The techniques used to extract bioactive fractions from seaweed are moving away from the conventional extraction methods [38] to options more in line with the concept of sustainability, such as subcritical water treatment [39], and ultrasound- or microwave-assisted extraction [17]. Based on the use of this extraction technology on the brown seaweed *S. latissima*, no results from other authors were found.

### 2.3. Features of Liquid Fractions

**Figure 2:**
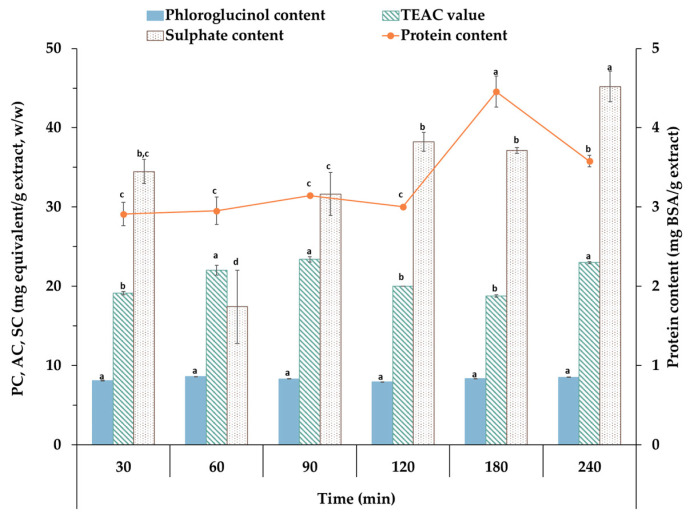
Total phloroglucinol content (PC), antioxidant activity in terms of TEAC values (AC), sulphate content (SC) and protein content for extracts from *S. latissima* processed under different durations of ultrasound (from 30 to 240 min). Note: n ≥ 3, letters in columns represent significant differences (*p* > 0.95).

The fractions recovered at different extraction times were analyzed, including phloroglucinol content, antioxidant activity as a TEAC value, sulphate content and protein content. The results are summarized in Figure 2.

The results regarding the sulphate content show the maximum was found at the longest time used, 240 min, with a value of 45.20 mg/g extract. This parameter rose significantly at the highest tested extraction temperatures. On the other hand, the highest value obtained for TEAC value was around 22–23 mg/g extract at 60 and 90 min, respectively, with no significant differences found. Regarding the phloroglucinol content, the value was similar for all the durations investigated, whereas the protein content reached its maximum at 180 min of ultrasound-assisted extraction. In this way, different fractions, with potential research interests in several industries, have been obtained. A work developed by Ummat and coauthors (2020) extracted bioactive compounds from *F. vesiculosus* using ultrasound assistance [40]. They found higher values of phlorotannin content, but, the extraction was performed using ethanol as a solvent at different percentages. These results could, therefore, be due to the influence of the solvent in the extraction. The content of sulphate was lower than in other brown seaweed extracts, particularly when enzymatic extraction was used, with values in the range of 20 to 40% (*w*/*w*) [41]. In another work, it was found that *S. latissima* showed the highest antioxidant activity, in comparison with *Palmaria palmata*. The ORAC values were studied, and it was found that the values of the antioxidant capacity for *P. palmata* were lower, in comparison with the values achieved by *S. latissima.* This behavior could have been due to the origin of the seaweeds; *P. palmata* was cultivated in controlled tanks, while *S. latissima* was cultivated in a marine farm (on submerged longlines) [42].

Protein content was also assessed, and the values obtained were around 3–4.5 mg of BSA per g of extract; this parameter increased significantly following processing at 180 °C, without statistical variation below this temperature. In another work, the values achieved were similar to those of the current work, from 3 to 5.5 mg/g extract approximately [43]. In a work where the extraction technology was different, the results were more than the current work: 9–12 mg/g extract [44]. This behavior could be due to the extraction process used or the seaweed selected. The composition of the seaweed is related to the origin of the algae, the collection season and other parameters [45].

**Figure 3:**
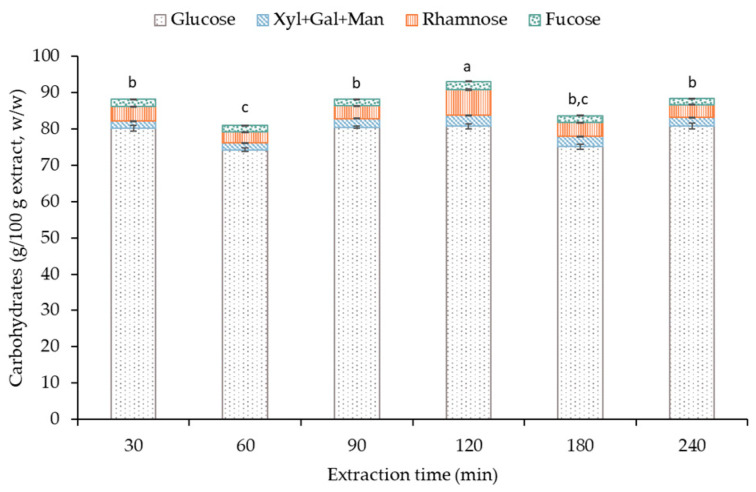
Carbohydrates content for extracts from *S. latissima* processed under different ultrasound (US) time conditions (from 30 to 240 min). Note: n ≥ 3, letters in columns represent significant differences (*p* > 0.95).

Another important parameter in the characterization of seaweed extracts is the carbohydrate composition; in this work, these values are summarized in Figure 3. In this case, the content of glucose was the largest component in all the fractions recovered. Moreira and coauthors (2023) applied different extraction methods to obtain the sugar composition of the fraction; in all cases and similar to the current work, the main saccharide was glucose [46]. As aforementioned, several parameters can influence the composition of the extract obtained by ecofriendly extraction technologies. In the current work, the content of glucose suggests a possible structural profile of the fucoidan of this seaweed based on glucose saccharide.

**Figure 4:**
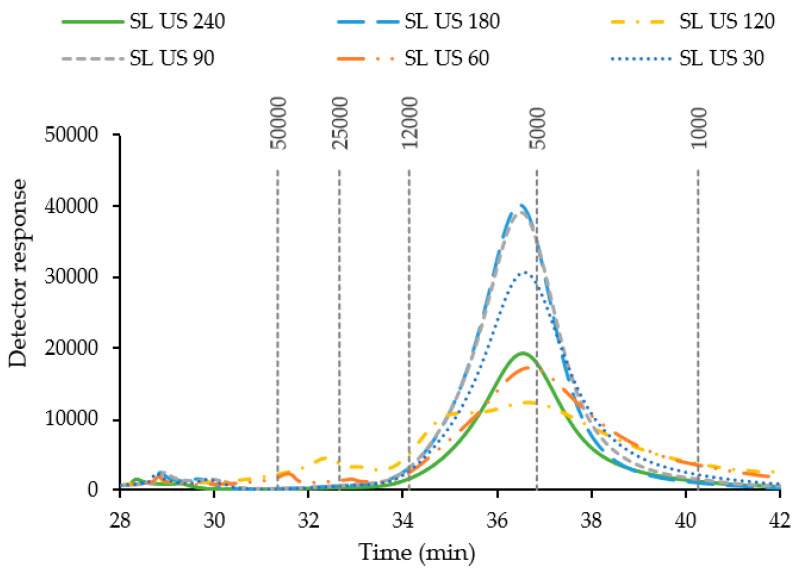
Molecular mass distribution for extracts from *S. latissima* (SL), processed under different ultrasound (US) time conditions (from 30 to 240 min). In the vertical lines are the values of the patterns, namely of dextran, from 1000 to 50,000 Da.

The molecular mass distribution is represented in Figure 4. In all cases, a main peak above the dextran pattern of 5000 g/mol was found. Also, slight peaks of the highest molecular weight, around the values of 25,000 and 50,000 g/mol, were found. The extraction process could be responsible for this profile behavior. According to these results, *S. latissima* is a seaweed with a highly accessible cell wall for the extraction of bioactive compounds and, furthermore, after ultrasound-assisted extraction, the main fractions exhibit relatively low molecular weight values. In a work by Rioux et al. (2010) with *S. longicruris*, they found that the extracted laminarin exhibited values between 2890 and 3320 Da, depending on the harvest period [47]. In another work, a similar value to the current work was found for a fraction of fucoidan extracted from *S. dentigera*: around 5 kDa [48]. These differences may be due to the extraction technology or methodology used, the severity of the treatment, or the operational conditions, as well as the harvest season and year of the seaweed selected [49].

**Figure 5:**
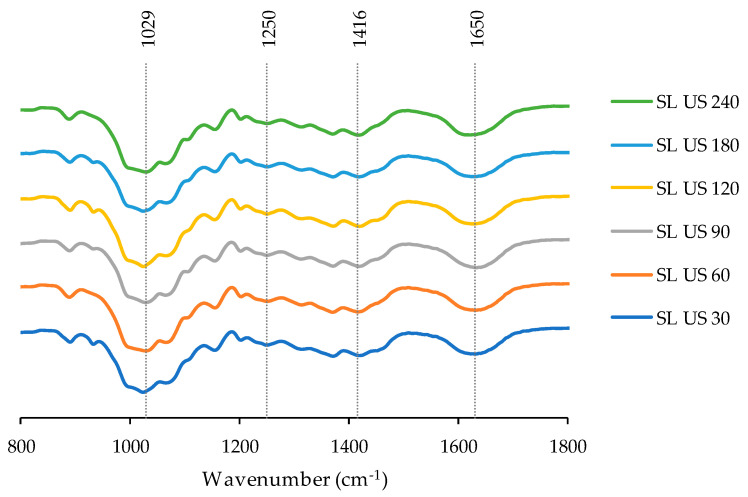
FTIR spectra for extracts from *S. latissima* (SL) obtained by ultrasound-assisted extraction (US) at different time conditions (from 30 to 240 min).

The Fourier-transform infrared spectroscopy (FT-IR) spectra are summarized in Figure 5. In all cases, the bands were registered in all the *S. latissima* extract samples studied. The band found at 1029 cm^−1^ was associated with the C-O group, whereas the peak at 1250 cm^−1^ was attributed to the asymmetric stretching vibration of the sulfate esters (O=S=O). The band found at 1416 cm^−1^ was attributed to the deformation vibration of C-OH, with the involvement of the symmetric stretching vibration of O-C-O, and the peak obtained at 1650 cm^−1^ was assigned to the carbonyl group of a carboxylic acid group [50,51]. Other authors found similar bands for extracts from *Saccharina japonica* [52]. No differences were observed in the peaks; this behavior indicates that the extraction process used was suitable to extract bioactive compounds from *S. latissima* while keeping the sulphate groups; this parameter is important because it is associated with the biological activities of the brown seaweeds [46].

### 2.4. Formulation and Production of Polymeric Microparticles

Based on the results of the characterization (Figure 2 and Figure 3) of the soluble fractions of *S. latissima*, the extract obtained at 240 min was selected to formulate the polymeric microparticles (MPs). As carriers, two polymers, alginate and mannitol (at 2%), were explored, as the value of production yield for both was similar, around 60% (*w*/*w*); this is summarized in Table 2. In the case of the loading capacity, mannitol achieved a value of 86%, higher than the value of alginate. In both cases, the values obtained for the production yield and loading capacity showed no statistical differences. It should be considered that the seaweed alginate fractions in the extract were not previously separated from the crude extract. In previous studies, the value of the alginate fraction after recovery could be around 10–15% [53]; this could influence the behavior of the microparticles formulated and their morphology. Other works exhibit how the temperature influences the degradation of the alginate, according to the rheological parameters; therefore, the highest temperature tested was selected in the current work to prepare the formulations [54].

Several factors could be influencing the formation process of the microparticles, as well as the concentration of the agent. Other authors found that a concentration of alginate of 1% produced non-spherical capsules, potentially due to an insufficient number of carboxyl groups, whereas a concentration above 5% increased the viscosity of the systems [55]. In another work, Łętocha and coauthors (2022) studied the type of physical methods that could be used to produce alginate microparticles, including spray drying, extrusion and emulsification/gelation [55].

**Figure 6:**
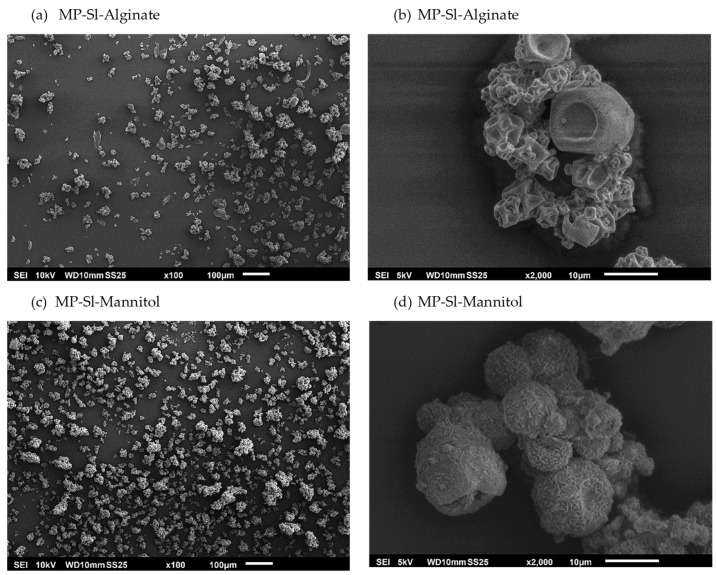
SEM images of the polymeric microparticles (MP) formulated with the extract obtained from *S. latissima* (SL) and two polymers: alginate (**a**,**b**) and mannitol (**c**,**d**), at different magnitudes (×100 and ×2000).

The shape and behavior of the polymeric microparticles were also studied by Scanning electron microscope (SEM) images and are exhibited in Figure 6. The images are representative of the formulations, and it was observed that the mannitol microparticles are more aggregated than the alginate microparticles. Differences in the shapes of the microparticles were also observed. In the case of the mannitol microparticles, they were spheric and rough, whereas the alginate microparticles had a concave surface. Also, a slight concave effect on the surfaces of the mannitol MPs was observed; this behavior may have been due to the presence of alginate in the crude extract used to formulate the MP. This effect on the surface of the microparticle could have also been due to the fast evaporation of the solvent during the drying of the solution to produce the particulate systems. Therefore, a concave surface could be expressed as an advantage because it decreases the permeability for gases and improves the protection of the bioactive compound [56]. Other works reporting on the use of these biopolymers have also shown particles exhibiting similar SEM images [57,58].

**Figure 7:**
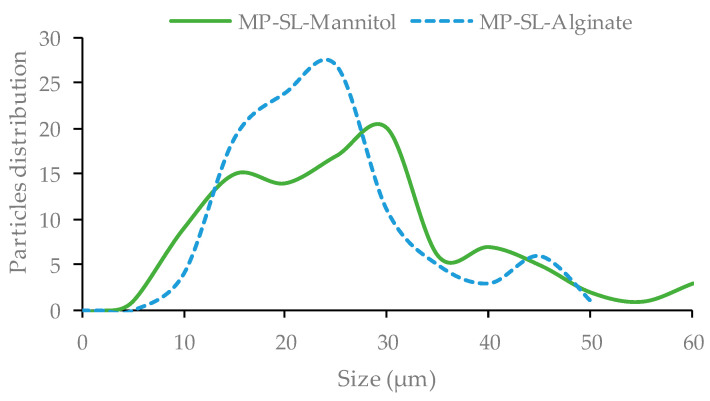
Profile of the particle distribution size of the microparticles (MP) formulated with the extract obtained from *S. latissima* (SL) at 240 min with two different polymers, mannitol and alginate.

Another parameter analyzed to evaluate the particles was the size profile of the microparticles formulated with the extract of *S. latissima* and the polymers (alginate and mannitol), shown in Figure 7. Differences associated with the polymer used were found. The microparticles formulated with mannitol exhibited a size between 20 and 25 µm, whereas the microparticles formulated with alginate showed two bands, around 15 µm and around 30 µm. This is a clear effect of the polymer used in the formulation, and both could display different behaviors in biomedical applications, for example with cell lines. An effect due to size differences could also probably be observed.

### 2.5. Thermorheological and Color Measurements

**Figure 8:**
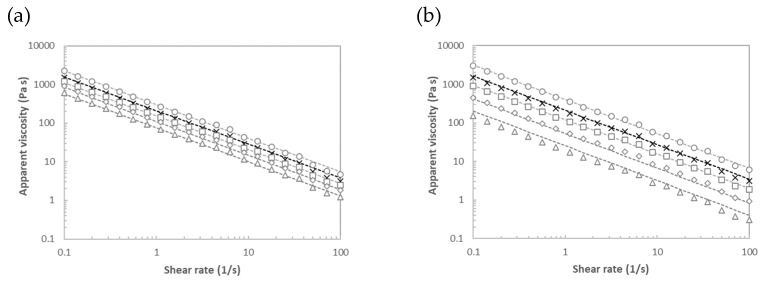
Rheological testing in terms of apparent viscosity versus shear rate of the proposed creams, incorporated with (**a**) mannitol or (**b**) alginate-based microparticles, at different tested temperatures: 5 (circles), 25 (squares), 35 (diamonds) and 45 (triangles) °C. Commercial cosmetic matrix is also plotted for comparative purposes (crosses). Lines correspond with the Ostwald–de Waele model.

Figure 8 shows the flow profiles of the proposed creams at different tested temperatures, incorporated with the (a) mannitol- and (b) alginate-based microparticles aforementioned. In all cases, the flow curves exhibited a typical shear-thinning behavior with a dramatical drop (of about 3 decades) in the apparent viscosity, with an increasing shear rate. At a fixed shear rate, the apparent viscosity decreased in the presence of microparticles, when compared with the commercial matrix tested at the same temperature. The magnitude of this parameter was consistent with those reported in the literature for similar matrices [32,33]. This behavior was more marked in the presence of alginate-based microparticles. In the same line, the creams made with alginate-based microparticles featured a more marked thermal effect. As expected, the apparent viscosity decreased with increasing temperature, since the resistance to flow is lower at the highest temperatures. It should be highlighted that no hysteresis effect was identified in any of the proposed systems. It was reported that drug release from microparticle-based topical gels can impact their bioavailability, efficacy and safety [59]. Later authors indicated that those matrices, when incorporated with microparticles of smaller sizes, led to faster drug release.

For all formulated creams, the experimental data of apparent viscosity (η) versus shear rate were successfully (R^2^ > 0.990) modelled according to the Ostwald–de Waele model (Table 3), written in terms of a model function for the viscosity flow curve [60]:

(1) $$\eta =k^{\prime }\gamma^{n-1}$$

where n (−) is the flow index and k′ (Pa s^n^) represents the flow consistency index. The flow index was practically invariant (between 0.10 and 0.13), which was consistent with those previously reported for biopolymer-based shear thinning systems [61].

**Figure 9:**
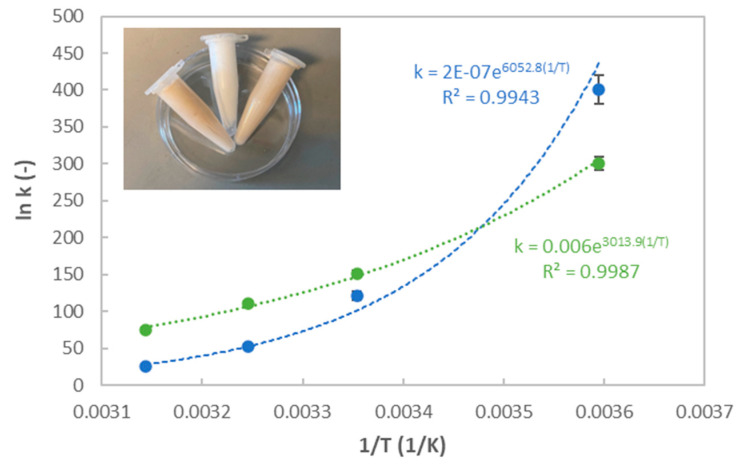
Thermal impact on the k parameter of the Ostwald–de Waele model, following a well-known Arrhenius expression (k = A exp (Ea/RT)), with A being the preexponential parameter, Ea being the activation energy and R being the ideal gas constant.

It should be highlighted that the k parameter increased with a decreasing temperature, exhibiting an Arrhenius behavior within the tested temperature range (Figure 9). This is consistent with the tendencies featured for other polymeric matrices, such as those formulated with guar or tragacanth gums at similar temperature ranges [61].

The proposed cosmetics, incorporated with microparticles, formulated with antioxidant compounds from *S. latissima* and using mannitol or alginate as a carrier, did not show water syneresis during six months of cold storage. Although this is a relevant industrial advantage, longer-term storability should be also assessed, since water release is an important problem that can take place after a few years of storage [62].

Concerning the color parameters, the proposed cosmetics independently of the incorporated microparticles did not show statistical differences in the color parameters. L* (93.4), a* (4.07) and b* (11.3) for the enriched creams exhibited larger values than those determined for the control commercial cream (L*: 99.1, a*: 0.55, b*: 0.19). Even though, the enriched creams exhibited small total color difference into the Adekunte et al. ranking (ΔE* < 1.5) [63].

## 3. Materials and Methods

### 3.1. Raw Material

Samples of *Saccharina latissima* were kindly provided by Portomuiños S.L. (A Coruña, Spain). The seaweed was dehydrated in an oven at 40–50 °C, until moisture levels were below 15%. The raw material was ground, until a particle size smaller than 0.5 cm, and was stored at room temperature in plastic bags in darkness.

### 3.2. Extraction Technology

Ultrasound-assisted extraction was the extraction technology selected. The working parameters were as follows: temperature at 80 °C (based on previous works [64,65]), duration between 30 and 240 min (at intervals of 30 min) and a solid:liquid ratio of 1:30 (*w*/*w*). The raw material was introduced in Pyrex flasks (250 mL) with distilled water. After the extraction, two fractions were recovered by filtration using a vacuum pump, a solid fraction and a crude liquid fraction; the latter was analyzed and used to formulate the polymeric microparticles.

### 3.3. Characterization of Raw Material

The moisture content analysis was performed gravimetrically, following the standard method developed by the Association of Official Analytical Chemists (AOAC, 1999, method 1) [66]. The ground sample was kept in an oven for 48 h at 105 ± 2 °C (P Theroven, JP Selecta, Barcelona, Spain). For the determination of the ash content, the sample was kept in a muffle furnace for 6 h at 575 °C (ELF, Carbolite, UK); it was then measured gravimetrically. The protein content was estimated using the Kjeldalh method, using the conversion factor (5.38) to determine the total nitrogen content [67].

#### 3.3.1. Minerals Content

The mineral content of the raw material was determined using several methods developed elsewhere [68]. In brief, 0.3 g of ash, 10 mL of nitric acid and 1 mL of hydrogen peroxide were mixed to perform an acidic digestion in a microwave (Marsxpress, CEM), operating at 200 W for 15 min before holding at 200 °C for 10 min. Ca, Fe, Cu and Mg were analyzed by atomic absorption spectroscopy, while Na and K were analyzed using atomic emission spectroscopy (SpectrAA-220 Fast Sequential from Varian, Palo Alto, CA, USA). Inductively coupled plasma mass spectrometry (ICP-MS) was used to determine the content of Cd (X Series, Thermo Scientific, Waltham, MA, USA).

#### 3.3.2. Oligosaccharide Fraction Determination

The oligosaccharide content of the raw material was analyzed based on a quantitative acid hydrolysis with sulfuric acid at 72%. Firstly, in a test tube, the sample and the sulfuric acid were mixed and kept in a water bath at 30 °C for 1 h (with manual stirring). Next, the sulfuric acid was diluted to 4% (*w*/*w*) concentration with distilled water, and the procedure continued in an autoclave (P-Selecta, Barcelona, Spain) at 121 °C for 1 h at 2 atm. The mixture was separated by filtration (using a vacuum pump) and the liquid phase was analyzed by high performance liquid chromatography (HPLC, 1260 Infinity, Agilent Technologies, Santa Clara, CA, USA), according to the method described elsewhere [53]. The solid phase (after filtration) was studied as an acid insoluble residue (AIR) and quantified gravimetrically.

### 3.4. Characterization of Liquid Fraction

The content of the extract of the liquid fraction or the soluble extract was calculated after drying 1 mL at 105 °C for 24–48 h in an air convective oven (P Theroven, P-Selecta, Barcelona, Spain).

The soluble protein content was studied following the method developed by Bradford [69]. The quantity of protein was analyzed according to the instructions specified by the supplier of the reagent. The standard used was bovine serum albumin (Sigma, Barcelona, Spain), and the absorbance was measured at 595 nm.

Glucose, rhamnose, fucose, xylose (xyl), galactose (gal) and mannose (man) (Sigma-Aldrich, Madrid, Spain) were quantified, according to the protocol described above. Previously, a hydrolysis of the liquid samples, using sulfuric acid at 4% concentration and an autoclave for 20 min at 121 °C, was performed.

Sulphate content was evaluated following the gelatin–barium chloride method [70]. This protocol was based on the hydrolysis of liquid samples using trichloroacetic acid (4%, Sigma-Aldrich, Madrid, Spain) to measure the sulphate content. Previously, gelatin–BaCl_2_ reagent was prepared: 0.5 g gelatin powder (Scharlau, Barcelona, Spain) was mixed with 100 mL of hot water (60–70 °C); when the gelatine was dissolved, the solution was kept at 4 °C for at least 6 h (or overnight). Next, 0.5 g BaCl_2_ (Sigma-Aldrich, Madrid, Spain) was added, resulting in a cloudy solution, and, after 2–3 h, the reagent was ready to use. Samples of distilled water as a control (0.1 mL), TCA solution (1.9 mL) and gelatin–BaCl_2_ reagent (0.5 mL) were introduced in a test tube, mixed in a vortex and incubated at room temperature for 15 min. The absorbance was read at 500 nm (Evolution 201, Thermo Scientific, Bremen, Germany), and the standard curve was performed with potassium sulphate (K_2_SO_4_) from 0.1 g/L to 2.0 g/L.

#### 3.4.1. Antioxidant Features

The phloroglucinol content (phenolic compounds in brown seaweeds) of the samples obtained were analyzed following the method developed by Koivikko et al. [71], which is a modification of the Folin–Ciocalteu method. The standard curve was performed using a solution of phloroglucinol from 0.030 g/L to 0.005 g/L. Next, the Folin–Ciocalteu reagent (VWR Chemicals, Rosny-sous-Bois, France) was diluted (1:1, *w*/*w*) with distilled water, and a solution of sodium carbonate solution (Sigma-Aldrich, St. Louis, MO, USA) at 20% concentration (*w*/*v*) in distilled water was prepared. In brief, 1 mL of sample, 1 mL of diluted Folin–Ciocalteu reagent and 2 mL of sodium carbonate were introduced in test tubes, homogenized in a vortex, and incubated for 1 h in darkness at room temperature. The absorbance was read at 730 nm using a spectrophotometer (Evolution 201, Thermo Scientific, Bremen, Germany). The results were represented as the equivalent mg of phloroglucinol per g of extract.

The antioxidant capacity was determined by a Trolox Equivalent Antioxidant Capacity assay, also known as a TEAC assay [72]. In brief, the liquid samples obtained after extraction (10 μL) were placed in a test tube, with distilled water for a control. The ABTS (2,2′-azino-bis(3-ethylbenzothiazoline-6-sulfonic acid) diammonium salt) solution, previously prepared, was diluted with PBS (phosphate-buffered saline) to attain an absorbance of 0.7 ± 0.1 at 734 nm. The ABTS diluted solution (1 mL) was added in the test tube above the liquid sample. Next, the test tubes were incubated for 6 min at 30 °C in a water bath. The absorbance of the samples was measured at 734 nm (spectrophotometer: Evolution 201, Thermo Scientific, Bremen, Germany). The standard curve was performed using Trolox (Sigma-Aldrich, Søborg, Denmark) and the results were expressed as TEAC values.

All above analyses were performed at least in triplicate.

#### 3.4.2. Structural Profiles

##### High Performance Size Exclusion Chromatography

The molar mass distribution profile of samples was studied by high performance size exclusion chromatography, using the HPLC previously described. Two columns were used in series, TSKGel G2500PW_XL_ and G3000PW_XL_ (300 × 7.8 mm, Tosoh Bioscience, Griesheim, Germany), and a PWX-guard column (40 × 6 mm, Tosoh Bioscience, Griesheim, Germany). The operational conditions were as follows: the mobile phase was 0.4 mL/min (Milli-Q water) and the column was working at 70 °C. Dextran at different molecular weights was the standard used (1000 to 80,000 g/mol, Fluka, St. Louis, MO, USA).

##### Fourier-Transform Infrared Spectroscopy

The liquid samples (lyophilized) and the polymeric microparticles were studied using the FTIR method. Firstly, the samples were blended with KBr and dried using an infrared lamp (30 min). The FTIR spectra were recorded at 800–1800 nm at 25 scans/min (Bruker IFS 28 Equinox equipment, OPUS-2.52, Billerica, MA, USA); for data acquisition, System 450-MT2 was used.

### 3.5. Formulation and Production of Polymeric Microparticles

The equipment to produce microparticles was a mini spray-dryer B-290 (BÜCHI, Flawil, Switzerland) equipped with a standard cyclone, and the nozzle was 1.5 mm. The operational parameter was the inlet temperature (103 °C, with the outlet around 72 °C), and the pump worked at a 100% feed solution flow rate.

Two polymers were evaluated to formulate the microparticles: alginate and mannitol. Both are biopolymers; in the case of mannitol, its high compatibility and inert properties have resulted in research interest for pharmaceutical applications.

#### 3.5.1. Characterization of Microparticles

##### Yield of Production

The production yield of the microparticles was determined gravimetrically (%, *w*/*w*), according to Equation (2):

(2) $$Production\;yield\;\left(\%\right)=\frac{mg\;microparticles\;recovered}{mg\;extract+mg\;mannitol}\times 100$$

##### Scanning Electron Microscopy

The shape and distribution of the polymeric microparticles formulated with alginate and mannitol were evaluated using SEM (JEOL JSM6010LA, Tokyo Japan). Previously, the particulate systems were covered by a gold layer (15 nm), and several images at different magnitudes were acquired.

##### Size Profile of Microparticles

SEM images were used to evaluate the size of the microparticles produced by applying the software, ImageJ, version 1.53 t. Over 100 measures were performed per formulation to represent the profile of the size.

### 3.6. Formulation of the Creams and the Corresponding Mechanical Measurements

The preparation of biocosmetics, based on a commercial cold cream (Guinama laboratories, Valencia, Spain), was studied. The proposed matrices were incorporated with the above selected antioxidant microparticles (2%, *w*/*w*). Samples were stored in the fridge (4–6 °C) until further analysis and were thermally tempered at room temperature for at least 60 min before rheological and color testing were undertaken.

#### 3.6.1. Flow Curves

Steady-state shear measurements of the proposed cosmetics were conducted at 5 °C, 25, 35 and 45 °C on a controlled stress rheometer (MCR 302 Paar Physica, Austria, Austria). A sand-blasted plate–plate geometry of 25 mm diameter (0.5 mm gap) was used for measuring geometry. The flow curves, in terms of apparent viscosity vs. shear rate of the selected cosmetics, were measured between 0.1 and 100 1/s. The possible presence of syneresis was also studied by monitoring the forward and backward flow curves. It should be indicated that systems were sealed with light silicon oil to avoid the drying of the matrices during thermorheological measurements. The cosmetics were rested for 10 min in the measuring geometry before rheological testing, to favor the structural and thermal equilibration of the samples [61].

#### 3.6.2. Water Syneresis

The water release of the cold stored systems was studied for 6 months, using the procedure previously indicated [73]. Briefly, the proposed cosmetics were located in test tubes in the fridge during the analyzed period. They were taken from the fridge weekly, centrifuged for 30 min at 500 rpm and weighed. The water release was calculated (%) as the water removed compared to the matrix’s initial weight.

#### 3.6.3. Color Testing

Color parameters (L*, a* and b*) were measured at least in fivefold within the CIELab space at room temperature, employing a Tristimulus colorimeter (Minolta CR-400, Tokyo, Japan). The L* parameter indicated the lightness, varying between 0 (whiteness) and 100 (brightness); a* described the greenness (a* < 0) or redness (a* > 0) degree; and b* indicated the blueness (b* < 0) and yellowness (b* > 0) level. The color differences (ΔE), compared to the commercial cosmetic in the absence of the microparticles, was also studied.

### 3.7. Statistical Analysis

All experiments were performed at least in triplicate. A statistical analysis of the experimental data was performed using a one-factor analysis of variance, ANOVA, employing the PASW Statistics software (v.22 IBM SPSS Statistics, New York, NY, USA). A post hoc Scheffé test was performed, to differentiate average values whenever the ANOVA study exhibited differences between means. A confidence degree of 95% (*p* < 0.05) was selected for the statistical analysis.

## Figures and Tables

**Table 1:** Proximal composition in dry basis (*w*/*w*) of the raw material *S. latissima*.

Fraction	%	Fraction	mg/kg
Moisture	10.98 ± 0.14	Minerals	
Ash	13.15 ± 0.02	Na	12,898.2
Protein	5.50 ± 0.26	K	22,175.1
Sulphate	1.38 ± 0.23	Mg	4838.0
AIR *	15.77 ± 0.34	Ca	9932.5
		B	85.4
Carbohydrates		Fe	112.2
Glucose	47.84 ± 0.48	Cu	3.1
Xylose	3.01 ± 0.09	Cd	0.02
Fucose	1.67 ± 0.04	Pb	0.02
Glucuronic acid	1.20 ± 0.05	Hg	0.01
		As	5.49

* AIR: acid insoluble residue; the minerals presented with standard deviations lower than 2.5%.

**Table 2:** Production yield of the polymeric microparticles formulated by adding 2% mannitol or alginate as carriers to liquid extract obtained by ultrasound-assisted extraction at 240 min from *Saccharina latissima* (SL).

MP SL 240	Mannitol	Alginate
Yield (%)	59.24 ± 1.12 ^a^	60.42 ± 1.68 ^a^
Loading capacity (%)	86.17 ± 1.28 ^a^	84.52 ± 1.55 ^a^

Letters in columns represent significant differences (*p* > 0.95).

**Table 3:** Fitting Ostwald–de Waele model parameters for the proposed creams.

Matrices	Cream with MP–SL–Mannitol			Cream with MP–SL–Alginate		
Parameters	k (Pa s^n^)	n (−)	R^2^	k (s^n^)	n (−)	R^2^
Control, 25 °C without MP	210.1 ± 0.3 ^b^	0.12 ± 0.01 ^a^	0.995	210.3 ± 0.1 ^b^	0.11 ± 0.01 ^a^	0.995
5 °C	300.7 ± 0.5 ^a^	0.13 ± 0.01 ^a^	0.992	402.8 ± 1.3 ^a^	0.11 ± 0.01 ^a^	0.991
25 °C	151.2 ± 0.4 ^c^	0.13 ± 0.01 ^a^	0.990	122.3 ± 0.9 ^c^	0.11 ± 0.01 ^a^	0.992
35 °C	110.1 ± 0.4 ^d^	0.12 ± 0.01 ^a^	0.993	53.2 ± 0.5 ^d^	0.10 ± 0.01 ^a^	0.990
45 °C	75.5 ± 0.1 ^e^	0.12 ± 0.01 ^a^	0.991	25.4 ± 0.6 ^e^	0.10 ± 0.01 ^a^	0.993

Data are given as average ± standard deviation. Letters in columns represent significant differences (*p* > 0.95).

## Data Availability

Data will be provided on request to the corresponding author.
